# Supplementary material for: Advances and Therapeutic Perspectives in Extended-Stage Small-Cell Lung Cancer
Source: Cancers (Basel). 2020 Nov 1;12(11):3224. doi: 10.3390/cancers12113224 (PMC7692868; doi:10.3390/cancers12113224)
Supplement: Supplementary file 1 [file cancers-12-03224-s001.pdf]

Table 1 supplementary data : Ongoing studies in ED-SCLC setting

Study	Phase	Setting	Interventional arm	Control arm
NCT04453930	II	1°L	camrelizumab + irinotecan/platinum followed by camrelizumab + Apatinib maintenance	NP
NCT04422210	I	1°L	venetoclax + atezolizumab + carboplatin/etoposide.	NP
NCT04346914	I	1°L	ZKAB001 + carboplatin/etoposide	NP
NCT04256421	III	1°L	Atezolizumab + carboplatin/etoposide + tiragolumab	atezolizumab + carboplatin/etoposide +placebo
NCT04254471	II/III	1°L	AL3810 + carboplatin/etoposide	carboplatin/etoposide + placebo
NCT03387332	I	1°L	APG-1252	NP
NCT04063163	III	1°L	HLX10 + carboplatin/etoposide	carboplatin/etoposide + placebo
NCT04028050	III	1°L	atezolizumab + carboplatin/etoposide	NP
NCT04012606	III	1°L	toripalimab + Platinum/etoposide	platin/etoposide + placebo
NCT03963414	I	1°L	carboplatin/etoposide + durvalumab followed by durvalumab maintenance	carboplatin/etoposide+durvalumab+tremelimumab followed by durvalumab maintenance
NCT04005716	III	1°L	tislelizumab+platin+etoposide	platin/etoposide + placebo
NCT03913455	II	1°L	guadecitabine and carboplatin	NP
NCT03850067	I/II	1°L	CC-90011+cisplatin/etoposide followed by CC-90011 or CC-90011+nivolumab+cisplatin/etoposide followed by CC-90011 and nivolumab	NP
NCT03841136	II	1°L	anlotinib + platinum/etoposide	NP
NCT03700359	II	1°L	anlotinib + lobaplatin + etoposide followed by anlotinib	lobaplatin + etoposide
NCT03568097	II	1°L	avelumab + platinum/etoposide	NP
NCT03387332	I	1°L	APG-1252	NP
NCT01441349	II	1°L	cisplatine + irinotecan + simvastatine	cisplatin + irinotecan
NCT02934503	II	1°L	platinum + etoposide + pembrolizumab and radiation	NP
NCT02722369	II	1°L	gemcitabine + carboplatine + hydroxychloroquine	carboplatin + etoposide
NCT02171325	II	1°L	irinotecan + cisplatin	NP
NCT03983759	II	maintenance	sintilimab	NP
NCT03923270	I	maintenance	thoracic radiotherapy + durvalumab or durvalumab+tremelimumab or durvalumab + olaparib	NP
NCT03958045	II	maintenance	platinum doublet + rucaparib and nivolumab	NP

NCT03830918	I/II	maintenance	niraparib + temozolomide	NP
NCT03769935	II	maintenance	cisplatin/etoposide + S1	cisplatin/etoposide
NCT04334941	II	maintenance	atezolizumab + talazoparib	atezolizumab
NCT03532880	I	maintenance	thoracic radiotherapy and olaparib	NP
NCT03516084	III	maintenance	niraparib	placebo
NCT03319940	I	maintenance	AMG 757 or AMG757 + Pembrolizumab	NP
NCT02402920	I	maintenance	pembrolizumab + radiation therapy	NP
NCT02566993	III	2°L	doxorubicin and lurbinectedin	CAV or topotecan
NCT03253068	II	2°L	pembrolizumab + amrubicin	NP
NCT03613753	II	2°L	irinotecan + lobaplatin	irinotecan
NCT03672773	II	2°L	temolozomide + talazoparib	NP
NCT03670056	II	2°L	nivolumab + ipilimumab	NP
NCT03904719	II	2°L	CM082 and JS001	NP
NCT03262454	II	2°L	atezolizumab	NP
NCT03227016	I/II	2°L	topotecan + veliparib	NP
NCT01737502	I/II	≥2°L	auranofin and sirolimus	NP
NCT02487095	I/II	≥2°L	VX-970 + topotecan	NP
NCT02446704	I/II	≥2°L	olaparib + temozolomide	NP
NCT02769962	I/II	≥2°L	CRLX101+olaparib	NP
NCT03216343	I	≥2°L	chiauranib	NP
NCT03088813	II/III	≥2°L	irinotecan hydrochloride liposome Injection	topotecan
NCT03009682	II	≥2°L	olaparib	NP
NCT03554473	I/II	≥2°L	M7824 or M7824 + topotecan or M7824 + temozolomide	NP
NCT03547804	II	≥2°L	apatinib	NP
NCT03406715	II	≥2°L	nivolumab + ipilimumab + Ad.p53-DC	NP
NCT03896503	II	≥2°L	topotecan + M6620	topotecan
NCT03879798	I/II	≥2°L	DS-3201b+irinotecan	NP
NCT04253145	I	≥2°L	PM01183 + Atezolizumab	NP
NCT04213937	II	≥2°L	nab-paclitaxel	topotecan
NCT04210037	I/II	≥2°L	APG-1252 + paclitaxel	NP
NCT04209595	I/II	≥2°L	PLX038 + rucaparib	NP
NCT04192682	II/III	≥2°L	Anlotinib + sintilimab	NP
NCT04173325	I	≥2°L	nivolumab + irinotecan followed by nivolumab maintenance	NP
NCT04128800	II	≥2°L	apatinib + S-1	NP

NCT04400188	I/II	≥2°L	fluzoparib + temozolomide or fluzoparib + temozolomide + SHR-1316	NP
NCT04381910	II	≥2°L	irinotecan hydrochloride liposome injection	NP
NCT04056949	II	≥2°L	IBI308 + paclitaxel/albumin paclitaxel	NP
NCT04055792	II	≥2°L	sintilimab + anlotinib	anlotinib
NCT04010357	II	≥2°L	abemaciclib	NP
NCT03994744	II	≥2°L	sintilimab + metformin	NP
NCT03823118	II	≥2°L	S1 + anlotinib	NP
NCT03732846	II	≥2°L	anlotinib	NP
NCT03728361	II	≥2°L	nivolumab + temozolomide	NP
NCT03639194	I	≥2°L	ABBV-011 + budigalimab	NP
NCT03575793	I/II	≥2°L	nivolumab + ipilimumab + plinabulin followed by nivolumab and plinabulin	NP
NCT03392064	I	≥2°L	AMG 119	NP
